# Identification of flocculant wine yeast strains with improved filtration-related phenotypes through application of high-throughput sedimentation rate assays

**DOI:** 10.1038/s41598-020-59579-y

**Published:** 2020-02-17

**Authors:** Cristian Varela, Caroline Bartel, Damian Espinase Nandorfy, Anthony Borneman, Simon Schmidt, Chris Curtin

**Affiliations:** 1 The Australian Wine Research Institute, PO Box 197, Glen Osmond, Adelaide, SA 5064 Australia; 2 Department of Wine & Food Science, University of Adelaide, Glen Osmond, SA 5064, Adelaide, Australia; 3 Present Address: College of Agricultural Sciences, Oregon State University, Wiegand Hall, 3051 SW Campus Way, Corvallis, OR 97331 USA

**Keywords:** Industrial microbiology, Industrial microbiology

## Abstract

In most yeast-driven biotechnological applications, biomass is separated from the aqueous phase after fermentation or production has finished. During winemaking, yeasts are removed after fermentation by racking, filtration, or centrifugation, which add costs to the overall process and may reduce product yield. Theoretically, clarification and filtration can be aided through use of yeast strains that form flocs due to cell-cell binding, a process known as flocculation. However, because early flocculation can cause stuck/sluggish fermentations, this phenotype is not common amongst commercially available wine yeasts. In this study we sought to identify wine strains that exhibit late-fermentation flocculant behaviour using two complementary approaches; a high-throughput sedimentation rate assay of individual strains and a competitive sedimentation assay using a barcoded yeast collection. Amongst 103 wine strains, several exhibited strong sedimentation at the end of the wine fermentation process under various environmental conditions. Two of these strains, AWRI1688 and AWRI1759, were further characterised during red winemaking trials. Shiraz wines produced with both strains displayed improved filtration-related properties. AWRI1759 produced wines with greater filterability, whereas AWRI1688 enabled the recovery of larger wine volumes after racking. Thus, this study demonstrates the effective use of sedimentation screening assays to identify wine yeasts with practical winemaking applications.

## Introduction

Cell-to-cell binding resulting in the formation of large clusters of cells that settle to the bottom of the fermentation vessel is known as flocculation. This behaviour involves a nonsexual, homotypic and reversible aggregation of yeast cells to form multicellular masses containing thousands of yeast cells^1–3^. After formation, these clusters (or flocs) separate rapidly from the medium by sedimentation, with larger particles settling faster than smaller cell aggregates^2,3^. The formation of flocs provides a benefit to yeast populations, where at least some cells may escape from harsh environmental conditions and survive nutrient starvation^4^. Several environmental variables have been described that affect flocculation, including; oxygen availability, temperature, pH, ethanol content and the concentration of fermentable sugars^3^.

Flocculation is relevant across several industrial yeast applications including; brewing, winemaking, bottle-fermented sparkling wine production, bioethanol production, bioremediation of heavy metals and the production of heterologous proteins^1,5^. In beer and wine production, several clarification strategies are usually performed to obtain a clear, stable product. Successful yeast flocculation facilitates clarification and downstream processing, simplifying enormously yeast removal from the final fermented product. This reduces the need for time-consuming and expensive cell removal methods such as centrifugation and filtration^1,6^. However, timing is a key consideration as flocculation should not occur before the fermentation itself has been completed. Premature loss of yeast from the bulk ferment can result in sluggish fermentations and may also lead to severe off-flavours^2^.

Yeast flocculation is highly complex in terms of phenotypes, signalling pathways, responsible genes and regulatory networks. Nevertheless, cell adhesion during flocculation can be broadly explained as a two-stage process that is initially driven by glycan-glycan interactions^7,8^ and then followed by glycan-lectin binding^9,10^. Lectin-like proteins, also known as flocculins, protrude from the cell walls of flocculent cells and bind to cell wall polysaccharides, notably mannans and glucans^1,2,9^. Flocculins are the products of the *FLO* gene family in *S. cerevisiae*, which can be subdivided into two groups^11^. The first group includes the genes *FLO1*, *FLO5*, *FLO9* and *FLO10*, which encode adhesins that promote cell-cell adhesion and contribute to the formation of multicellular clumps^1,2,9,12^. The second group includes the genes *FIG2.* and *AGA1* which are induced during mating^13,14^, along with *FLO11* which encodes a protein responsible for cellular adhesion to substrates, pseudohyphae formation and invasive growth^15–17^. An additional member of the family, *FLO8* encodes a transcriptional activator of *FLO1*, *FLO9*, *FLO11* and *STA1*, which encodes an extracellular glucoamylase^2,12^.

**Figure 1:**
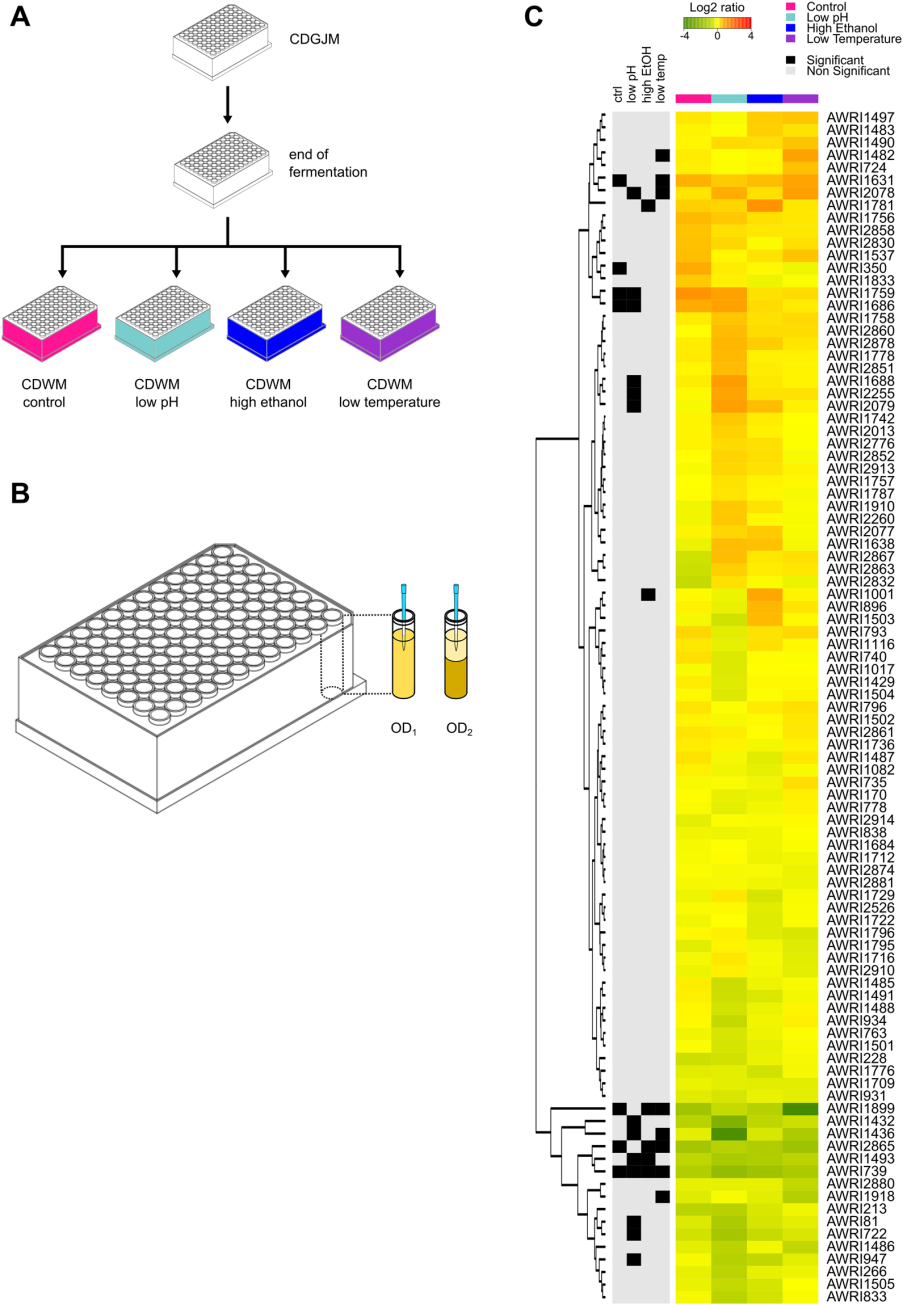
Sedimentation rate assay of individual strains. (**A**) After fermenting CDGJM, individual strains were centrifuged and resuspended CDWM with varied composition. Control (pH 3.5, ethanol 12.5% v/v, 28 °C); low pH (pH 3.0, ethanol 12.5% v/v, 28 °C); high ethanol (pH 3.5, ethanol 15.5% v/v, 28 °C); and low temperature (pH 3.5, ethanol 12.5% v/v, 12 °C (**B**) After cultures were adjusted to a similar optical density, OD_600_ was determined twice. For the second reading wells were sampled after 20 mins just below the liquid surface. Sedimentation rate was calculated as the difference between the two OD_600_ measurements divided by the incubation time. (**C**) Heatmap showing the Log2 ratio between the sedimentation rate of a given strain and the mean for all strain under a particular condition. Red indicate strains which sediment faster than the mean, whereas green indicate strains which sediment slower than the mean.

Two main flocculation phenotypes have been described for *S. cerevisiae*, taking into consideration differences in reversible inhibition of flocculation by sugars, salt, and low pH, and the relative sensitivity of flocs to protease activity. For yeast strains exhibiting the “Flo1” phenotype, flocculation is inhibited by mannose, whereas the “NewFlo” phenotype is inhibited by low pH, glucose, maltose, mannose and sucrose but not galactose, and is sensitive to digestion by trypsin or proteinase K^1^. Both Flo1 and NewFlo phenotypes are Ca^2+^-dependent and can be attributed to *FLO1*-, *FLO5*- and *FLO9*- overexpression in *S. cerevisiae* strains^15,18–22^. Two additional minor flocculation phenotypes include a mannose insensitive (MI) phenotype^23^, and, a phenotype in which flocculation is induced by ethanol^24^.

In a winemaking context, the ideal wine yeast should exhibit robust flocculation that manifests at the end of fermentation^1,25^. However, only a small proportion of wine yeasts are naturally flocculant^26,27^. Several genetic engineering approaches have therefore been employed to improve yeast flocculation^19,28,29^. Here we utilised two complementary sedimentation assays to study the flocculation behaviour of 103 wine yeast strains. A high-throughput sedimentation rate assay of individual strains and a competitive sedimentation rate assay using a barcoded yeast collection enabled us to identify strains with strong sedimentation properties at the end of the wine fermentation process. Selected strains were then evaluated for filtration-related phenotypes (i.e. filterability, residual lees volume) and their effects on the sensory properties of Shiraz wine.

## Results

### Sedimentation rate assay of individual strains

The sedimentation rate of 95 yeast strains (Table S1) was determined with a high-throughput assay using deep 96-well plates. After fermenting Chemically Defined Grape Juice Medium (CDGJM), strains were centrifuged and resuspended in Chemically Defined Wine Medium (CDWM) under four different environmental conditions: control, low pH, high ethanol and low temperature (Fig. 1). Several strains showed enhanced sedimentation under different environmental conditions. Four strains showed increased sedimentation under control conditions (pH 3.5, ethanol 12.5% v/v, 28 °C), seven for low pH (pH 3.0, ethanol 12.5% v/v, 28 °C), two for high ethanol (pH 3.0, ethanol 15.5% v/v, 28 °C) and two for low temperature (pH 3.0, ethanol 12.5% v/v, 12 °C). AWRI1631, AWRI1686, AWRI1759 and AWRI2078 showed enhanced sedimentation for more than one condition. In contrast, 10 strains showed reduced sedimentation under several conditions with AWRI739 exhibiting decreased sedimentation for all conditions (Fig. 1C).

**Figure 2:**
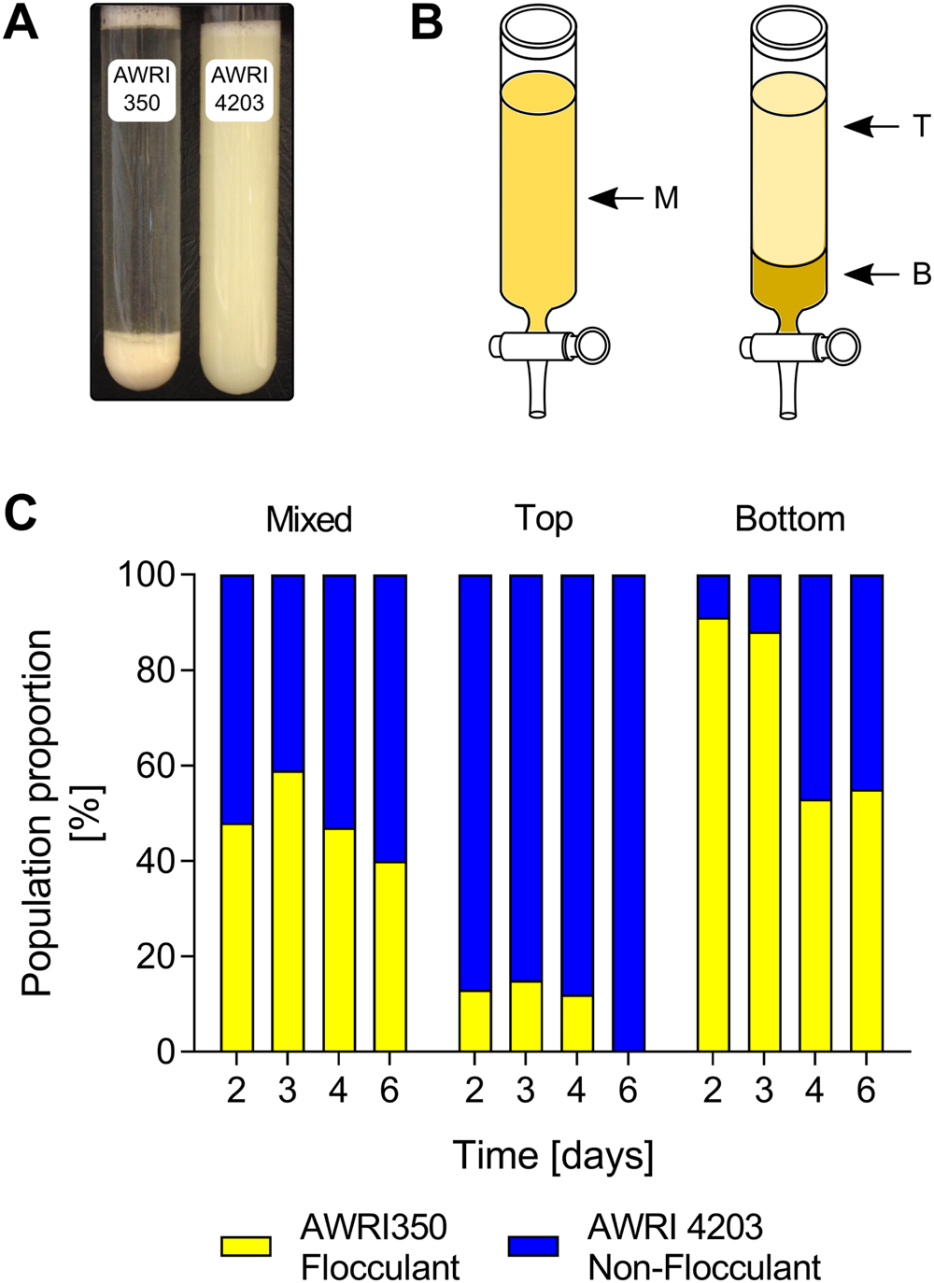
Preliminary mixed culture sedimentation experiment. (**A**) Flocculation after 20 minutes of incubation time for flocculent strain AWRI350 and non-flocculant derivative AWRI4203 in CDGJM. (**B**) At different times during fermentation, mixed cultures of AWRI350 and AWRI4203 were poured into a glass column fitted with a glass tap at the bottom. Samples were taken before the culture settled (M, mixed), and after 20 minutes of incubation from the top (T) and the bottom (**B**) of the settled culture. (**C**) Population proportions for AWRI350 and AWRI4203 in mixed, top and bottom samples during fermentation.

### Preliminary mixed culture sedimentation experiment

In order to evaluate the feasibility of analysing sedimentation behaviour in a mixed-culture environment, a proof-of-concept trial was initiated based upon a simple binary system. Two *S. cerevisiae* strains were therefore used in mixed cultures; AWRI350 (highly flocculant) and AWRI4203 (non-flocculant derivative of AWRI350 carrying an antibiotic marker) (Fig. 2A). At different times during fermentation, individual and mixed cultures of AWRI350 and AWRI4203 were assessed for sedimentation efficiency using a glass column. Samples were taken before settling (M, mixed), and after 20 minutes of incubation from both the top (T) and the bottom (B) of the column (Fig. 2B). Before settling, mixed cultures showed roughly similar proportions for both strains throughout the fermentation (Fig. 2C). After settling, samples taken from the top of the culture consisted predominantly (>85%) of the non-flocculant strain AWRI4203. In contrast, samples taken from the bottom of the culture contained a high proportion of the flocculant strain AWRI350 (>88%) for the first 3 days of fermentation. These samples contained similar cell concentrations of both strains at later stages of fermentation (Fig. 2C), indicating co-flocculation of both strains over time. Nevertheless, these results suggested that given the different composition for flocculant/non-flocculant strains in the three evaluated positions within the column, it was feasible to progress to more complex mixed cultures for high-throughput screening.

### Competitive sedimentation rate assay

**Figure 3:**
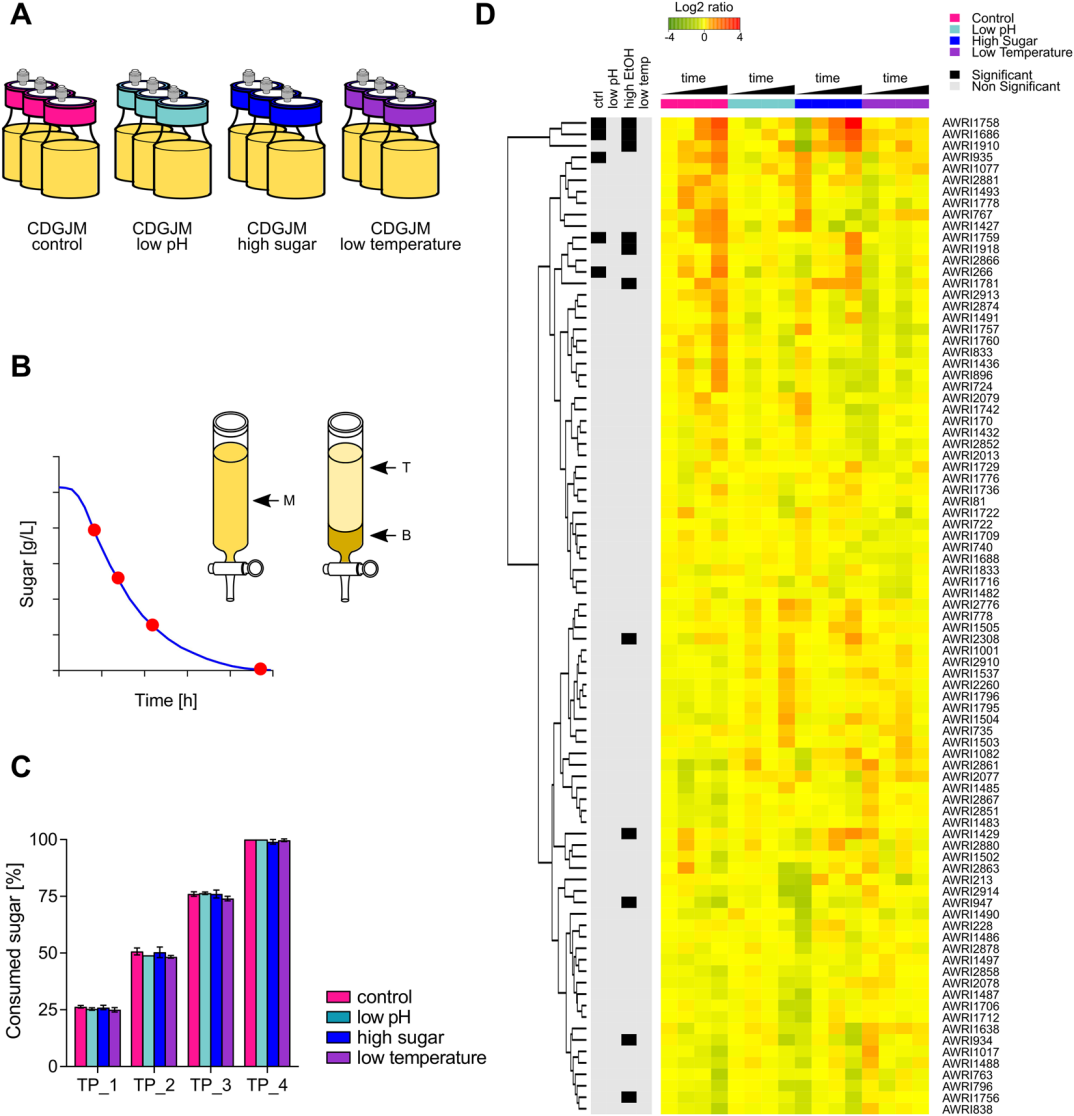
Competitive sedimentation rate assay. (**A**) A barcoded yeast collection was inoculated in triplicate in CDGJM and allowed to ferment under four different conditions: control (sugar 200 g/L, pH 3.5, 28 °C); low pH (sugar 200 g/L, pH 3.0, 28 °C); high sugar (sugar 260 g/L, pH 3.5, 28 °C); and low temperature (sugar 200 g/L, pH 3.5, 12 °C). (**B**) Cultures were sampled at four time-points (TP) during fermentation and poured into a glass column fitted with a glass tap at the bottom. Samples were taken before the culture settled (M, mixed), and after 20 minutes of incubation from the top (T) and the bottom (**B**) of the settled culture. (**C**) Consumed sugar at each of the four time-points for all conditions. (**D**) Heatmap showing the Log2 ratio between bottom and top samples during fermentation under different conditions. Red indicate strains present in the bottom of the culture, whereas green indicate strains present in the top of the culture.

Given the limitations imposed by the limited set of antibiotic markers available in yeast, a DNA-based barcode strategy was implemented to allow for higher-order multiplexing^30^. This mixed collection of 89 barcoded wine yeast strains (Table S1) was used in competitive sedimentation rate assays across four different environmental conditions: control, low pH, high sugar and low temperature (Fig. 3, Table S2). Resultant wine chemical composition was similar between the control, low pH and low temperature conditions (Table 1), whereas residual sugar, glycerol and ethanol concentrations were different for ferments performed under high sugar conditions relative to the control.

**Table 1:** Final composition of wines produced with a barcoded yeast library under different environmental conditions.

	Control	Low pH	High sugar	Low temperature
Residual sugar [g/L]^#^	0.0 ± 0.0	0.0 ± 0.0	3.2 ± 2.2^***^	0.7 ± 0.5
Glycerol [g/L]	8.7 ± 0.2	8.6 ± 0.2	10.3 ± 0.1^**^	8.3 ± 0.1
Acetic acid [g/L]	0.2 ± 0.0	0.5 ± 0.0	0.5 ± 0.1	0.3 ± 0.1
Malic acid [g/L]	3.1 ± 0.2	2.6 ± 0.0	2.8 ± 0.1	3.2 ± 0.1
Succinic acid [g/L]	0.4 ± 0.0	0.3 ± 0.0	0.5 ± 0.0	0.3 ± 0.0
Ethanol [% v/v]	12.3 ± 0.2	12.0 ± 0.1	15.2 ± 0.1^***^	11.5 ± 0.7

^#^Residual sugar includes glucose and fructose.

Stars indicate statistically significant differences compare to the control condition (^**^p < 0.01, ^***^p < 0.001).

Similar to the preliminary mixed culture sedimentation experiment, barcoded mixed cultures were assayed at different times during fermentation via differential sedimentation in a glass column. Samples were then taken before the culture settled (M, mixed), and after 20 minutes of incubation from the top (T) and the bottom (B) of the settled culture (Fig. 3B). Sugar consumption at each of the four time-points was not statistically different between conditions (Fig. 3C). Comparison between top and bottom samples showed an enrichment for nine strains in the cell sediment, with five strains showing increased sedimentation for the control condition and seven for the high sugar condition (Fig. 3D). All of these showed increased sedimentation only at the end of fermentation. AWRI1686, AWRI1758 and AWRI1759 showed enhanced sedimentation for more than one condition. Only three strains were enriched in the culture supernatant, AWRI934, AWRI947 and AWRI1756, all under the high sugar condition.

### Confirmation experiments for individual strains

Three strains, AWRI1686, AWRI1759 and AWRI1781, showed increased sedimentation in both assays for at least one environmental condition (Table S3). In contrast, only AWRI947 showed decreased sedimentation in both assays. Of the 79 strains evaluated in both assays, only one strain showed conflicting results. AWRI1918 exhibited a decreased sedimentation rate at low temperature in the individual cultures assay, and increased sedimentation at high sugar in the mixed culture assay (Table S3).

Several strains were then selected to confirm their flocculation behaviour under different environmental conditions as individual cultures. In addition to the three strains identified in both sedimentation assays three strains that showed enhanced flocculation in only one assay, AWRI1482, AWRI1688 and AWRI1758, were also included. AWRI1482 showed increased sedimentation at low temperature, while AWRI1688 exhibited enhanced sedimentation at low pH, both were identified in the individual cultures assay. AWRI1758 showed increased sedimentation under control and high sugar conditions in the mixed culture assay. These six strains (AWRI1482, AWRI1686, AWRI1688, AWRI1759, AWRI1758 and AWRI1781) are located in different branches of the phylogenetic tree described by Borneman *et al*.^31^, which was constructed using the genome sequences of 212 *S. cerevisiae* strains (Fig. S1). AWRI739, which showed reduced sedimentation in all conditions was used as reference strain for confirmation experiments.

**Figure 4:**
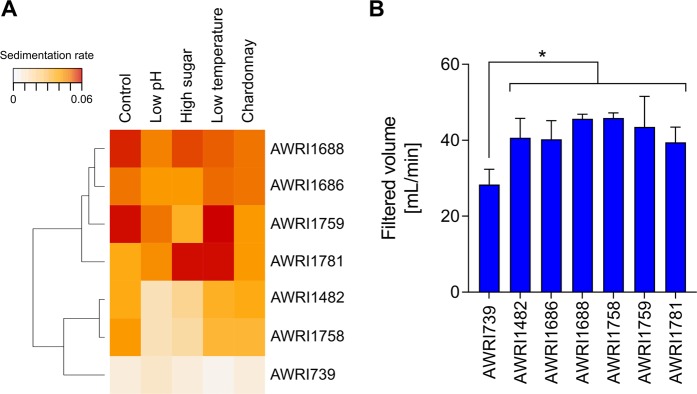
Confirmation experiments for individual strains. (**A**) Sedimentation strains for selected individual strains under five different conditions. (**B**) Filterability for Chardonnay wines produced with selected individual strains. Star indicates statistically significant differences (p < 0.05).

All strains showed faster sedimentation rate than the reference strain in at least three conditions (Fig. 4A). Two groups of fast settling strains were identified. The first group, AWRI1686, AWRI1688, AWRI1759 and AWRI1781, settled more rapidly in all conditions. A second group displayed a higher sedimentation rate than the reference strain under control and low temperature CDGJM conditions, and in Chardonnay juice. AWRI1688 and AWRI1759 exhibited strong sedimentation under control conditions, whereas AWRI1759 and AWRI1781 showed particularly fast sedimentation rates at low temperature.

A faster and stronger sedimentation rate can result in a more efficient wine clarification process, which in turn may facilitate easier wine filtration. To evaluate if this was the case, selected strains were used to produce Chardonnay wines, which were then tested for filterability. Filtration rate was significantly greater for wine made with all tested strains compared to wines made using the reference strain (Fig. 4B).

### Winemaking trial

**Figure 5:**
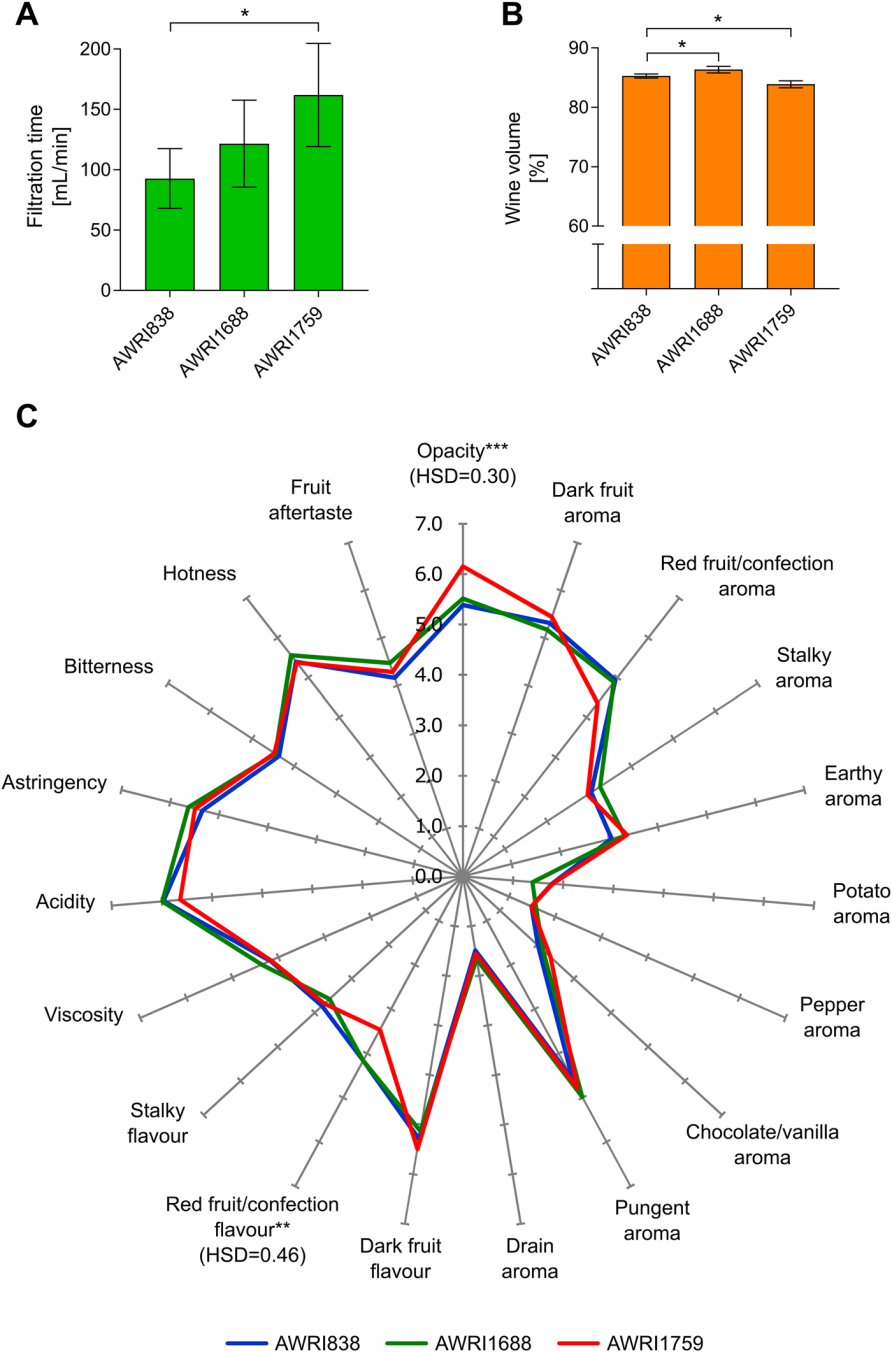
Winemaking trial. (**A**) Filtration time, (**B**) wine volume after racking, for Shiraz wines produced with AWRI838, AWRI1688 and AWRI1759. Statistically significant differences are indicated with stars (* p < 0.05). (**C**) Mean sensory attribute intensity scores for Shiraz wines, significant levels are indicated with stars (*p < 0.05, **p < 0.01, ***p < 0.001).

In order to evaluate if selected flocculant strains could improve the filterability of wine made at pilot scale, a winemaking trial using Shiraz must was performed. Two strains, AWRI1688 and AWRI1759, were selected for this trial. AWRI1759 showed increased sedimentation in both screening assays and in confirmation experiments, while AWRI1688 was the only commercially available wine strain which showed enhanced sedimentation rate in any test. AWRI838, a representative of widely used commercial strains (PDM subclade, see Fig. S1), was used as a control for the trial. The chemical composition of all resultant wines was similar (Table 2). Following standardised winemaking practices, filterability of racked wines produced with AWRI1759 was significantly improved, achieving a filtration rate almost double that for wines produced with AWRI838 (Fig. 5A). Furthermore, the exponential fouling constant was also lower in wines produced with AWRI1759 (4.35 m^−1^) relative to control wines (10.63 m^−1^) indicating a lower concentration of colloid fractions in wines produced with this strain. Interestingly, the volume of wine recovered after racking was 1% greater in wines produced with AWRI1688 compared to AWRI838 wines, whereas the volume recovered for AWRI1759 wines was 1.3% lower than the AWRI838 wines (Fig. 5B).

**Table 2:** Final composition of Shiraz wines.

	AWRI838	AWRI1688	AWRI1759
Residual sugar [g/L]^#^	0.1 ± 0.0	0.1 ± 0.0	0.1 ± 0.0
pH	3.6 ± 0.0	3.5 ± 0.0	3.5 ± 0.0
Titratable acidity [g/L]	5.5 ± 0.1	6.3 ± 0.1	5.9 ± 0.3
Glycerol [g/L]	10.4 ± 0.0	10.3 ± 0.2	10.4 ± 0.2
Acetic acid [g/L]	0.2 ± 0.0	0.3 ± 0.0	0.3 ± 0.0
Malic acid [g/L]	0.0 ± 0.0	0.0 ± 0.0	0.0 ± 0.0
Succinic acid [g/L]	0.9 ± 0.1	0.8 ± 0.0	0.7 ± 0.1
Ethanol [% v/v]	15.6 ± 0.1	15.7 ± 0.0	15.8 ± 0.1
Free SO_2_ [mg/L]	44 ± 1	45 ± 2	43 ± 3
Total SO_2_ [mg/L]	136 ± 12	135 ± 20	164 ± 27^***^

^#^Residual sugar includes glucose and fructose.

Stars indicate statistically significant differences compare to the control condition (^**^p < 0.01, ^***^p < 0.001).

The sensory profile of the resulting wines revealed minor differences between the different yeast strains (Fig. 5C). Of the 19 sensory attributes selected by the panel to describe the wines, only two, opacity (colour intensity) and red fruit/confection flavour, were rated significantly different among the strains. Wines produced with AWRI1759 showed higher opacity and lower red fruit/confection aroma and flavour than the other wines, while wines produced with AWRI1688 did not significantly differ from those made using AWRI838 in any sensory attributes rated.

## Discussion

Yeast flocculation is of substantial importance for the wine industry, since it can provide an effective, simple and cost-effective way to separate yeast biomass from wine at the end of fermentation. Two complementary approaches were employed to evaluate the flocculation behaviour of 103 wine yeast strains. The first approach involved a high-throughput assay based on monitoring optical density change to determine sedimentation rate, while the second employed a barcoded wine yeast collection to measure the enrichment of strains during fermentation in the cell sediment and the supernatant after a defined period of settling.

Before using the second approach, a preliminary mixed culture sedimentation experiment was conducted to evaluate the feasibility of running competitive sedimentation assays in mixed cultures. While the preliminary experiment showed enrichment of a non-flocculant strain in samples taken from the top of the culture after settling throughout fermentation, cell sediment samples showed co-flocculation of flocculant and non-flocculant strains later in fermentation. Co-flocculation has been observed between non-flocculant *S. cerevisiae* strains^32^, between non-flocculant and flocculant strains of *S. cerevisiae*^33^, and between *S. cerevisiae* and non-*Saccharomyces* yeast strains^6,33^. This process can be strain and species specific and relates to interactions between cell membrane components, most likely encoded by the *FLO* genes^6,33^. During batch culture, the composition and architecture of the yeast cell wall changes as a result of changing environmental conditions^34^. Factors such as ethanol concentration and temperature can affect the cell surface hydrophobicity (CSH), which in turn can trigger flocculation^3^, while lipid unsaturation, that can be a result of hypoxia, can induce *FLO1* expression causing flocculation^35^. All these factors can explain the co-flocculation of flocculant and non-flocculant strains observed later in fermentation during the preliminary mixed culture sedimentation experiment. Nevertheless, the preliminary experiment indicated that it was possible to evaluate yeast flocculation in mixed cultures given the enrichment of flocculant and non-flocculant strains in the different positions within a settled culture. Additionally, this experiment enabled evaluation of early sedimentation onset, which is a critical factor since flocculation should not occur before the fermentation itself has been completed.

Environmental factors known to have an effect on yeast flocculation, such as pH, temperature, ethanol content and sugar concentration^3^, were evaluated in both sedimentation assays. While the high-throughput assay revealed significant differences in flocculation behaviour in all conditions, the competitive sedimentation assay only showed significant differences for two conditions, control and high sugar concentration. Since the latter assay is based on relative enrichment in different positions within a column after culture settling, it is possible that co-flocculation, as observed during the preliminary mixed-culture experiment, affected the outcome for the other environmental conditions. In fact, enrichment can be seen for some strains at the end of fermentation for the low pH condition although this was not statistically significant (Fig. 3).

Both, the high-throughput sedimentation rate assay and the competitive sedimentation rate assay showed good complementarity, identifying several strains that flocculated strongly during fermentation, and strains exhibiting poor sedimentation behaviour under the evaluated conditions. These different groups of strains are likely explained by the great genetic diversity of the *FLO* genes in wine yeasts^22^. In fact, the *FLO* genes show high copy number variation, with *FLO5* and *FLO11* among the 60 genes with highest copy number variation in the yeast genome^36^. *FLO* genes, particularly *FLO1* and *FLO5*, also show diverse gene expression profiles^37^ which may or may not be linked to copy number. Specific variations in the amino acid sequence of Flo proteins, particularly in *FLO11*, have also been shown to influence yeast flocculation^38^. In addition, the concentration of lectin-like cell surface receptors correlates with yeast flocculation ability^9^.

Six strains showing flocculation behaviour under different conditions, three strains identified in both sedimentation assays, two in the high-throughput assay, and one in the competitive mixed culture assay, were selected for confirmation experiments. All six strains were able to strongly sediment at the end of fermentation in CDGJM and Chardonnay juice. All strains produced wine with improved filterability. During wine filtration small colloidal components in wine, as well as pectins, can adhere to the filter surface causing them to foul rapidly increasing filtering costs^39,40^. Commercial enzyme preparations, as well as *Saccharomyces* and non-*Saccharomyces* yeast strains exhibiting relevant enzyme activities can be used to improve wine filterability^40,41^. It is likely that the membrane composition of the selected flocculant strains enable interaction not only with other cells but also with other wine components during post-fermentation settling, therefore enhancing filterability as they reduce the concentration of these components

Two of the selected strains, AWRI1688 and AWRI1759, were used in red wine pilot-scale trials along with a widely used commercial yeast, to evaluate filterability, lees density and sensory profile. AWRI1688 was the only commercially available wine strain which showed enhanced sedimentation rate, a phenotype that is not common amongst commercial wine yeasts. Whereas AWRI1759 showed increased sedimentation in all tests. Sensory descriptive analysis of the resulting wines revealed only minor differences between the different yeast strains, which is not surprising given their relative positions in the *S. cerevisiae* phylogenetic tree (Fig. S1). Several empirical fouling indices and the maximum volume of filtrate have been employed to estimate wine filterability, however all these are poorly correlated with the soluble colloid content^39^. Nevertheless, the exponential fouling constant, estimated from mathematical modelling of the filter resistance, is a good measure of filter fouling for membranes and it is well correlated with the unstable colloids that affect filtration^39^. Wines produced with AWRI1759 showed higher filterability and a smaller exponential fouling constant than control wines, indicating a lower concentration of colloid fractions in AWRI1759 wines. Although a correlation between strong flocculation, in genetically engineered yeasts, and turbidity has been reported previously^20^, our results directly link efficient yeast flocculation and enhanced wine filterability. These results suggest, therefore, that the use of AWRI1759 should result in enhanced filtration efficiency and likely lower filtration costs for winemakers.

Interestingly, the volume of lees in wines produced with AWRI1688 was lower than in control wines, which in turn, resulted in a larger volume of wine recovered after racking. Govender *et al*.^20^ observed very compacted lees fractions in wines produced with transgenic yeast strains suggesting that this could increase wine recovery. Our results also demonstrate that yeast flocculation can increase wine yield through reducing losses. Although a 1% increase in wine volume may seem small, when considered in the context of wine tanks that may have capacities in excess of 500 hL, this would generate an extra volume of at least 500 L of wine when using AWRI1688. In summary, this study demonstrates the effective use of complementary sedimentation screening assays to identify wine yeasts with practical applications that can benefit winemakers significantly.

## Material and Methods

### Yeast strains and media

*S. cerevisiae* and *S. cerevisiae* hybrid strains used in this study are listed in Table S1. All strains were obtained from the Australian Wine Research Institute (AWRI) Wine Microorganism Culture Collection (WMCC). Cryogenically preserved (−80 °C) strains were cultured and maintained on YM plates (3 g/L malt extract, 3 g/L yeast extract, 5 g/L peptone, 10 g/L glucose, 16 g/L agar) and stored at 4 °C. YPD medium (20 g/L glucose, 20 g/L peptone, 10 g/L yeast extract) was used to grow pre-inocula cultures. Chemically Defined Grape Juice Medium (CDGJM) emulating the composition of a white grape juice consisted of (per litre): glucose 100 g, fructose 100 g, citric acid 0.2 g, malic acid 3 g, potassium hydrogen tartrate 2.5 g, K_2_HPO_4_ 1.1 g, MgSO_4_.7H_2_O 1.5 g, CaCl_2_.2H_2_O 0.4 g, H_3_BO_3_ 0.04 g, proline 0.84 g, nitrogen as ammonium and amino acids to 307 mg N/L of yeast assimilable nitrogen (YAN), trace elements stock solution 1 mL, vitamins solution 1 mL, fatty acids stock solution 1 mL, and sterol stock solution 1 mL as previously described^42^. Chemically Defined Wine Medium (CDWM) emulating the composition of wine was prepared by fermenting CDGJM with *S. cerevisiae* AWRI1631 until all sugar was consumed. After fermentation CDWM was incubated at 4 °C for 5 days to separate the yeast biomass from the finished wine. Ethanol concentration and pH were then adjusted in the clarified wine to 12.5% v/v and pH 3.5 (control), 12.5% v/v and pH 3.0 (low pH) and 15.5% v/v and pH 3.5 (high ethanol), using pure ethanol and 10 M NaOH, respectively, and then filter sterilised (0.22 µm, Millipore).

### High-throughput sedimentation rate assay of individual strains

Although standard sedimentation tests have been used to evaluate yeast flocculation for many years^43,44^, these utilise conditions that differ significantly from those found during wine fermentation and which can affect cell-to-cell interactions. For this reason, yeast sedimentation was evaluated in wine fermentation conditions. The sedimentation rate of 95 yeast strains (Table S1) was evaluated as indicated below under four different environmental conditions: control (pH 3.5, ethanol 12.5% v/v, 28 °C); low pH (pH 3.0, ethanol 12.5% v/v, 28 °C); high ethanol (pH 3.5, ethanol 15.5% v/v, 28 °C); and low temperature (pH 3.5, ethanol 12.5% v/v, 12 °C). Strains were grown in 96-well plates containing 200 μL of YPD and incubated overnight at 28 °C. These cultures were then used to inoculate triplicate deep 96-well plates containing 1 mL of CDGJM. Plates were incubated at 28 °C for 7 days. A random subset of 15 strains were confirmed to complete fermentation within this timeframe. After fermentation, plates were centrifuged at room temperature for 5 minutes at 1000 × g, cultures resuspended in 1 mL of CDWM with varied composition as described above, and incubated for 24 hours at either 28 °C or 12 °C. A high-throughput assay was then used to determine sedimentation rate. Briefly, plates were agitated, an aliquot was then diluted (1:4) and used to measure optical density (OD_600_ nm) in standard 96-well plates using a Freedom EVO robotic liquid handling unit and a Tecan Infinite M200 spectrophotometer (Tecan Group Ltd., Switzerland). Based on these values, cultures were then adjusted to an OD_600_ of 1 in a new deep 96-well plate using the corresponding CDWM. Plates were then subsampled into a 96-well plate for OD_600_ nm measurement. After incubating 20 mins, deep well plates were resampled just below the liquid surface and OD_600_ nm determined. Sedimentation rate was then calculated as the difference between the two OD_600_ measurements divided by the incubation time. This method was calibrated with AWRI350 a highly flocculant yeast strain. Thus, sedimentation rate was indicative of yeast falling out of suspension due to enhanced flocculation behaviour under the different environmental conditions.

### Preliminary mixed culture sedimentation experiment

*S. cerevisiae* AWRI350 a highly flocculant yeast strain and AWRI3878 a non-flocculant derivative of AWRI350, were used to evaluate the feasibility of sedimentation assays in mixed cultures. AWRI3878 was obtained following the same procedure as described by Varela *et al*.^45^. Briefly, a 80 mL sample from the end of fermentation of CDGJM was allowed to settle at room temperature for 20 minutes. A 1 mL sample was then taken from the top of the settled culture and used to inoculate a flask containing 200 mL of sterile CDGJM. After a third selection step, samples from the top of settled cultures were plated and single colonies isolated. AWRI3878 was then transformed with a natMX cassette encoding clonNAT resistance creating strain AWRI4203. The cassette was PCR amplified from plasmid pAG25 (EURSCARF collection) using primers, inYKL-AG25-F and inYKL-AG25-R, containing 50 bp flanking regions (Table S4) which enabled integration in the intergenic region between YKL061W and YKL060W. Yeast transformation was performed using the lithium acetate/polyethylene glycol method^46^. Transformed strains were confirmed by PCR as described previously^47^.

Individual cultures of AWRI350, AWRI4203 and a mixed culture of both strains were inoculated at 1 × 10^6^ cells/mL in 100 mL Schott bottles fitted with one-way air valves and containing 80 mL of CDGJM. Four bottles were inoculated for each condition. Sacrificial cultures were sampled 2, 3, 4 and 6 days after inoculation. For each sample the whole culture was poured into a glass column fitted with a glass tap at the bottom, and allowed to settle at room temperature for 20 minutes. Subsamples were taken before the culture settled (M), from the top of the settled culture (T) and from the bottom (B). These were spread on YPD and YPD-clonNAT plates to determine total and AWRI4203 populations, respectively.

### Barcoded yeast library and yeast strain pools

A barcoded yeast library consisting of 89 yeast strains (Table S1), created as described elsewhere^30^, was used for competitive sedimentation assays. Barcoded strains contained a 150 base pair barcode comprised of a LoxP scar flanked by a molecular tag at each end and priming sites homologous to the sequencing primers used on Illumina HiSeq platforms. Barcodes were integrated into the *HO* locus of each strain and verified by individually sequencing across the *HO* region. Wine Yeast Barcoded (WYBC) pools were obtained as described previously^30^.

### Competitive sedimentation rate assay

A barcoded yeast collection (as described above) was used to compare the flocculation behaviour of yeasts during fermentation under four different environmental conditions: control (sugar 200 g/L, pH 3.5, 28 °C); low pH (sugar 200 g/L, pH 3.0, 28 °C); high sugar (sugar 260 g/L, pH 3.5, 28 °C); and low temperature (sugar 200 g/L, pH 3.5, 12 °C). CDGJM was modified accordingly to meet these conditions. WYBC pools were inoculated at a rate of 1 mL per 100 mL of CDGJM (approximately OD_600_ 0.1) in 100 mL Schott bottles fitted with one-way air valves and containing 80 mL of the corresponding CDGJM. Fifteen bottles were inoculated for each environmental condition corresponding to 5 sacrificial sample time-points in triplicate. Thus, sacrificial cultures were sampled at 0, 25, 50, 75 and 100% sugar consumption. For each sample the whole culture was poured into a glass column (length 20 cm, diameter 2.5 cm) fitted with a glass tap at the bottom, and allowed to settle at room temperature for 20 minutes. Subsamples were taken from three different positions within the column: before the mixed culture settled (M), from the top (T) and from the bottom (B) of the settled culture. Subsamples were centrifuged, and the resulting cell pellets were frozen at −80 °C. These were later used for custom-amplicon sequencing as described below. Subsamples taken before the culture settled were also used to monitor fermentation progression.

### Generation of amplicons from mixed cultures

Genomic DNA was extracted from cell pellets using a Gentra Puregene Yeast/Bact kit (Qiagen, Germany) as described previously^30^. Sequencing ready amplicons were generated through PCR using primers Illum_P5_S50(1–8) + Illum_P7_N70(1–12) (Table S4) containing P5 and P7 indexes, respectively^30^. Purified amplicons were then sequenced (HiSeq. 2500 single ended 150 bp reads) at Ramaciotti Centre for Genomics (NSW, Australia).

### Statistical analysis of sequencing reads

The change in sequencing counts (frequency of observation) for any given barcode (strain) was compared between the mixed culture (M), and the top (T) of the settled culture; the mixed culture, and the bottom (B) of the settled culture; and between the top and the bottom of the settled culture (positions within the column) for each environmental condition. Sequencing frequency (counts per barcode) for each indexed sample were used as the raw input to determine changes in yeast strain frequency. Statistical evaluation was done using EdgeR^48^ in R version 3.3.2^49^ as described previously^30^. Data analysis and graphical representation were further aided using the package ggplot2^50^. After analysis, two strains showed little or no information (samples with less than 15 reads) and were not considered further.

### Confirmation experiments for individual strains

Selected strains, AWRI739, AWRI1482, AWRI1686, AWRI1688, AWRI1758, AWRI1759 and AWRI1781, were tested individually in five conditions: control, low pH, high sugar, low temperature CDGJM; and filtered sterilised Chardonnay grape juice (250 g/L sugar, pH 3.34, 338 mg N/L yeast assimilable nitrogen (YAN), 7.4 g/L titratable acidity at pH 8.2). Triplicate flasks fitted with airlocks and containing 100 mL of media were inoculated at 1 × 10^6^ cells/mL and incubated with gentle shaking (120 rpm) at the corresponding temperature. At the end of the fermentation, sedimentation rate was determined using the high-throughput assay described above.

Individual strains were also used to evaluate if they produced wines that were easier to clarify and filter due to enhanced flocculation properties. For this, individual strains were inoculated in triplicate at 1 × 10^6^ cells/mL in 1 L Schott bottles fitted with one-way air valves and containing 900 mL of Chardonnay grape juice. Cultures were incubated at 22 °C with gentle agitation and fermentation kinetics monitored by weight loss. When fermentation finished wines were cold settled at 4 °C for 5 days, clarified wines were then transferred to new bottles and used to measure filterability as described below.

### Filterability test

The filterability test measures the change in flow rate over time when a wine is forced through a 0.45 μm membrane filter at constant pressure. For this an automated, bench-top measurement system similar to the system described by Alarcon-Mendez and Boulton^39^ was used. For the test, a minimum of 500 mL of each wine was introduced in a stainless-steel pressure chamber which is connected to a filter housing through a solenoid valve. Nitrogen is used to pressurise the chamber (2 bar), the valve is then opened and the volume of wine passing through the filter is recorded over time. The volume of filtered wine is related to the turbidity of the wine. In addition, mathematical models were used to estimate filter resistance (R) using an exponential equation (R = R_m_*exp[b*V/A]. R_m_ is the medium resistance, b is the exponential fouling constant, V is the volume of filtrate and A is the filter area. The exponential fouling constant is a good measure of filter fouling for membranes and it is well correlated with the unstable colloids that affect filtration, especially for commercial red wines^39^.

### Shiraz winemaking trial

Two strains, AWRI1688 and AWRI1759, were evaluated in winemaking trials using AWRI838, a widely used commercial yeast strain, as a control. Shiraz grapes obtained from the McLaren Vale region in 2017 (South Australia) were used to prepare must. This contained 270 g/L of sugar (equal amounts of glucose and fructose), 184 mg N/L of yeast assimilable nitrogen (YAN) and 3.6 g/L titratable acidity (determined to pH 8.2) with a pH of 3.69. Fermentations were performed in triplicate at 22 °C in fermentation vessels containing 50 kg of randomised grapes and 50 mg/kg potassium metabisulfite, which were stored at 15 °C overnight prior to inoculation, as described previously^45^.

Yeast starter cultures were grown overnight in YM medium under aerobic conditions at 28 °C, shaking at 120 rpm. These cultures were then used to inoculate 1 L of sterile Shiraz, diluted 1:1 with water, in 5 L Erlenmeyer flasks. Flasks were incubated overnight at 22 °C with shaking (120 rpm) under aerobic conditions and then used to inoculate Shiraz must. Ferments were incubated at 22 °C and the solids cap was plunged twice daily. Fermentation kinetics were followed by measuring density. After sugar was completely consumed, Shiraz wines were inoculated for malolactic fermentation (MLF) with *Oenococcus oeni* (Lalvin VP41, Lallemand) as recommended by the manufacturer. After MLF was completed, finished wines were sampled to determine basic chemical composition and to measure filterability as described above. Wine and lees (settled solids after fermentation) volumes were estimated by weight difference before and after racking. Before filtering and bottling, free SO_2_ was adjusted to 35–45 mg/L. Wines were bottled in 375 mL bottles under a screwcap closure and store for at least 6 weeks before analysis.

### Analytical techniques

Glucose, fructose, glycerol and organic acids were quantified by high-performance liquid chromatography (HPLC) using a BioRad HPX87H column as described previously^51^. Alcohol, pH and titratable acidity were determined by using a Foss WineScan FT 120 as described by the manufacturer (Foss, Hillerød, Denmark). Free and total SO_2_ were measured by the aspiration method^52^.

### Sensory descriptive analysis

Shiraz wines were evaluated by a panel of twelve assessors (nine females, three males) with an average age of 46 years (SD = 10.7). All panellists were part of the external AWRI trained descriptive analysis panel and had extensive experience in wine sensory descriptive analysis. Over four two-hour training sessions wines from the study were progressively used to generate and refine appropriate descriptive attributes and definitions through a consensus based approach^53^. The final list of attributes, definitions/synonyms and reference standards are shown in Table S5. Wine sample evaluation was performed as described previously^45^ with some modifications. Samples were presented to panellists in 30 mL aliquots in 3-digit-coded, covered, ISO standard wine glasses at 22–24 °C, in isolated booths under daylight-type lighting. All samples were expectorated. During the two formal data collection days, nine wines (three yeast strains x three fermentation replicates) were assessed in duplicate and presented in randomised modified Williams Latin Square incomplete block design generated by Fizz sensory acquisition software (version 2.46, Biosystemes, Couternon, France). Wines were assessed in sets of three wines per tray and assessors were forced to take a 30 second rest between samples and a minimum ten-minute rest between trays. Each presentation replicate was poured from a new bottle. The intensity of each attribute was rated using an unstructured 15 cm line scale numbered from 0 to 10, with indented anchor points of ‘low’ and ‘high’ placed at 10% and 90% respectively. Data was acquired using Fizz sensory software. (version 2.51, Biosystemes, Couternon, France). Panel performance was assessed using Fizz software and R with the SensomineR (sensominer.free.fr/) and FactomineR (factominer.free.fr/) packages. The performance assessment included analysis of variance for the effect of judge, wine and presentation replicate and their interactions, degree of agreement with the panel mean, degree of discrimination across samples and the residual standard deviation of each judge by attribute. All judges performed to an acceptable standard.

### Statistical analysis of chemical and sensory data

Statistical analysis was performed as described previously^45^. Briefly, differences between compositional measurements were determined using two-way ANOVA and Tukey’s multiple comparisons test with the software GraphPad Prism v6.03. For sensory data ANOVA and Tukey’s Honest significant difference (HSD) test were carried out using Minitab 18 (Minitab Inc., Sydney, NSW). The effects of yeast strain, judge, fermentation replicate nested in yeast strain, presentation replicate nested in yeast strain and fermentation replicate as well as the interactions between judge and yeast, and between judge and fermentation replicate nested in yeast were assessed. Judge was considered as a random effect. Differences were considered significant when *p* values were lower than 0.05.

## Supplementary information

Supplementary Figure .

Supplementary Table 1.

Supplementary Table 2 .

Supplementary Information.
