# Supplementary figures and images for: Identification of flocculant wine yeast strains with improved filtration-related phenotypes through application of high-throughput sedimentation rate assays
Source: Sci Rep. 2020 Feb 17;10:2738. doi: 10.1038/s41598-020-59579-y (PMC7026045; doi:10.1038/s41598-020-59579-y)

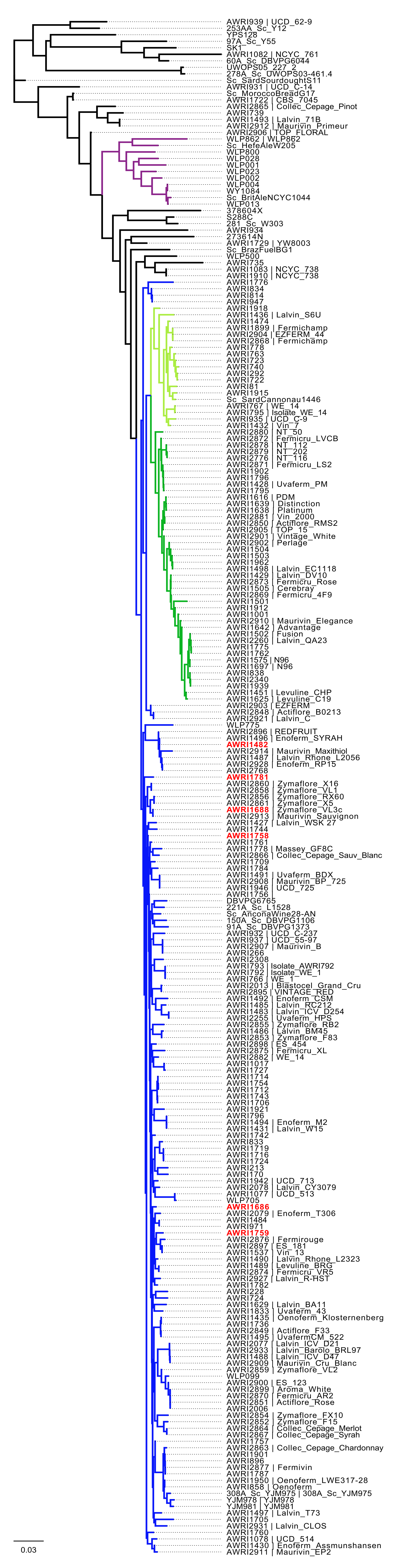

Supplement: Supplementary file 1 — Supplementary Figure . [file 41598_2020_59579_MOESM1_ESM.png]
