# Supplementary material for: Identification of flocculant wine yeast strains with improved filtration-related phenotypes through application of high-throughput sedimentation rate assays
Source: Sci Rep. 2020 Feb 17;10:2738. doi: 10.1038/s41598-020-59579-y (PMC7026045; doi:10.1038/s41598-020-59579-y)
Supplement: Supplementary file 4 — Supplementary Information. [file 41598_2020_59579_MOESM4_ESM.docx]

**Figure S1.** Phylogenetic tree for *S. cerevisiae* strains isolated from, or used in ale brewing (purple) or winemaking (blue and green). Dark- and light-green have been used to designate strains belonging to two main subclades within the wine yeasts. Dark green indicates the PDM subclade. Strains in red indicate selected strains for confirmation experiments. Adapted from Figure 2 Borneman et al. ^31^.

**Table S3.** Summary for strains showing increased or decreased sedimentation in individual and/or mixed culture assays.

| Strain | Sedimentation | Conditions | Assay |
| --- | --- | --- | --- |
| AWRI81 | decreased | low pH | individual |
| AWRI266 | increased | control | mixed |
| AWRI350 | increased | control | individual |
| AWRI722 | decreased | low pH | individual |
| AWRI739 | decreased | control, low pH, high ethanol, low temperature | individual |
| AWRI934 | decreased | high sugar | mixed |
| AWRI935 | increased | control | mixed |
| AWRI947 | decreased | low pH, high sugar | Individual, mixed |
| AWRI1001 | increased | high ethanol | individual |
| AWRI1429 | increased | high ethanol | mixed |
| AWRI1432 | decreased | low pH | individual |
| AWRI1436 | decreased | low pH, low temperature | individual |
| AWRI1482 | increased | low temperature | individual |
| AWRI1493 | decreased | low pH, high ethanol | individual |
| AWRI1631 | increased | control, low temperature | individual |
| AWRI1686 | increased | control, low pH, high sugar | Individual, mixed |
| AWRI1688 | increased | low pH | individual |
| AWRI1756 | decreased | high sugar | mixed |
| AWRI1758 | increased | control, high sugar | mixed |
| AWRI1759 | increased | control, low pH, high sugar | Individual, mixed |
| AWRI1778 | increased | low pH | individual |
| AWRI1781 | increased | high ethanol, high sugar | Individual, mixed |
| AWRI1899 | decreased | control, high ethanol, low temperature | individual |
| AWRI1918 | decreased | low temperature | individual |
| AWRI1918 | increased | high sugar | mixed |
| AWRI2078 | increased | low pH, low temperature | individual |
| AWRI2079 | increased | low pH | individual |
| AWRI2255 | increased | low pH | individual |
| AWRI2308 | increased | high sugar | mixed |
| AWRI2865 | decreased | control, high ethanol, low temperature | individual |

**Table S4.** Primers used in this study.

| Primer | Sequence (5’ – 3’) |
| --- | --- |
| inYKL-AG25-F | caaaagatgagctaggcttttgtaaaaatatcttacgttgtaaaattttaagcttgccttgtccccgccgg |
| inYKL-AG25-R | ttttcttctgaaggtcaatgacaaaatgatatgaaggaaataatgatttctcgacactggatggcggcgtt |
| Illum_P5_S501 | aatgatacggcgaccaccgagatctacactagatcgcacactctttccctacacgacgctcttccgatct |
| Illum_P5_S502 | aatgatacggcgaccaccgagatctacacctctctatacactctttccctacacgacgctcttccgatct |
| Illum_P5_S503 | aatgatacggcgaccaccgagatctacactatcctctacactctttccctacacgacgctcttccgatct |
| Illum_P5_S504 | aatgatacggcgaccaccgagatctacacagagtagaacactctttccctacacgacgctcttccgatct |
| Illum_P5_S505 | aatgatacggcgaccaccgagatctacacgtaaggagacactctttccctacacgacgctcttccgatct |
| Illum_P5_S506 | aatgatacggcgaccaccgagatctacacactgcataacactctttccctacacgacgctcttccgatct |
| Illum_P5_S507 | aatgatacggcgaccaccgagatctacacaaggagtaacactctttccctacacgacgctcttccgatct |
| Illum_P7_N701 | caagcagaagacggcatacgagattaaggcgagtgactggagttcagacgtgtgctcttccgatct |
| Illum_P7_N702 | caagcagaagacggcatacgagatcgtactaggtgactggagttcagacgtgtgctcttccgatct |
| Illum_P7_N703 | caagcagaagacggcatacgagataggcagaagtgactggagttcagacgtgtgctcttccgatct |
| Illum_P7_N704 | caagcagaagacggcatacgagattcctgagcgtgactggagttcagacgtgtgctcttccgatct |
| Illum_P7_N705 | caagcagaagacggcatacgagatggactcctgtgactggagttcagacgtgtgctcttccgatct |
| Illum_P7_N706 | caagcagaagacggcatacgagattaggcatggtgactggagttcagacgtgtgctcttccgatct |
| Illum_P7_N707 | caagcagaagacggcatacgagatctctctacgtgactggagttcagacgtgtgctcttccgatct |
| Illum_P7_N708 | caagcagaagacggcatacgagatcagagagggtgactggagttcagacgtgtgctcttccgatct |
| Illum_P7_N709 | caagcagaagacggcatacgagatgctacgctgtgactggagttcagacgtgtgctcttccgatct |
| Illum_P7_N710 | caagcagaagacggcatacgagatcgaggctggtgactggagttcagacgtgtgctcttccgatct |
| Illum_P7_N711 | caagcagaagacggcatacgagataagaggcagtgactggagttcagacgtgtgctcttccgatct |
| Illum_P7_N712 | caagcagaagacggcatacgagatgtagaggagtgactggagttcagacgtgtgctcttccgatct |

Underlined sequences indicate flanking regions for insertion in YKL locus

**Table S5.** Attributes, definitions and reference standards evaluated by panelists for Shiraz wines.

| **Attribute** | **Definition/Synonyms** | **Standard** |
| --- | --- | --- |
| ***Appearance*** |  |  |
| Opacity | Colour intensity, the degree of which light does not pass through the sample |  |
| ***Aroma*** |  |  |
| Dark fruit | Intensity of the aroma of dark fruits and berries: blackberries, plums, blueberries and blackcurrant | 2 x frozen blueberries, 1 x frozen blackberry (Sara Lee), 1 mL blackcurrant syrup (Ribena), and 3 g of plum paste (Maggie Beers) |
| Red fruit/Confection | Intensity of the aroma of red fruits, red confection and berries: raspberries, cranberries, strawberries and floral/musk lolly | 2 x frozen raspberries (Sara Lee), 1 frozen strawberry (Welch’s) and 1 µL of 2.11 g/L β-ionone |
| Stalky | Intensity of the aroma of green stalks, capsicum and rhubarb stalks | 5 µL of 53.82 mg/L of 3-isopropyl-2-methoxypyrazine |
|  |  |  |
| Earthy | Intensity of aroma earthiness, beets and mushrooms | 50 µL of 4 mg/L geosmin. |
| Boiled Vegetables/ Potato | Intensity of the aroma of vegetables and boiled potato | 20 µL of 1.5 mg/L methional |
| Pepper/ Spice | Intensity of the aroma of pepper and spice | 20 µL of 1.5 mg/L rotundone and 10 µL of 110 mg/L eugenol |
|  |  |  |
| Chocolate/ Vanilla | Intensity of the aroma of chocolate and vanilla | Standard 1. 10 µL of 1.00 mg/L vanillin  Standard 2. 3 chocolate chips (Absolute Organics) not in wine |
| Pungent | Intensity of the aroma and effect of alcohol. | 4 mL of 95% food grade ethanol (Tarac Technologies) |
| Drain | Intensity of the aroma of dirty drain, natural gas and boiled eggs | 20 µL of 2 % mercaptoethanol v/v and 0.01 g wood ash |
|  |  |  |
|  |  |  |
| ***Palate*** |  |  |
| Dark fruit | Intensity of the flavour of various dark fruits: blackberries, plums, blueberries and blackcurrants |  |
| Red fruit/Confection | Intensity of the flavour of red fruits, red confectionary and berries: raspberries, cranberries, strawberries and floral/musk lolly |  |
| Stalky | Intensity of the aroma of green stalks, capsicum and rhubarb stalks |  |
| Viscosity | The perception of the body, weight or thickness of the wine in the mouth. Low = watery, thin mouth feel. High = oily, thick mouth feel. | 1.5 g/L carboxymethylcellulose sodium salt (Sigma Aldrich) in water |
| Acidity | Intensity of acid taste in the mouth including aftertaste. | 2 g/L L-(+)-tartaric acid (Chem-Supply) in water |
| Astringency | The drying and mouth-puckering sensation in the mouth. Low = coating teeth; Medium = mouth coating & drying; High = puckering, lasting astringency. | 0.43 g/L alum sulfate (Ajax fine Chem Supply PTY LTD in water |
| Bitterness | The intensity of bitter taste perceived in the mouth, or after expectoration. | 0.15 g/L Quinine sulfate (Sigma Aldrich) in water |
| Hotness | The intensity of alcohol hotness perceived in the mouth, after expectoration and the associated burning sensation. Low = warm; High = hot, burning. | 8% food grade alcohol (Tarac Technologies) in water |
| Fruit AT | The lingering fruit flavour perceived in the mouth after expectorating. |  |

All red aroma standards were added to 30 mL of 2017 Yalumba premium selection bag-in-box Shiraz
